# *Galleria mellonella* experimental model for bat fungal pathogen *Pseudogymnoascus destructans* and human fungal pathogen *Pseudogymnoascus pannorum*

**DOI:** 10.1080/21505594.2018.1518087

**Published:** 2018-10-05

**Authors:** Beth Burgwyn Fuchs, Sudha Chaturvedi, Rodnei Dennis Rossoni, Patricia P de Barros, Fernando Torres-Velez, Eleftherios Mylonakis, Vishnu Chaturvedi

**Affiliations:** a Division of Infectious Diseases, Rhode Island Hospital, Warren Alpert Medical School at Brown University, Providence, RI, USA; b Mycology Laboratory, Division of Infectious Diseases, Wadsworth Center, New York State Department of Health, Albany, NY, USA; c Department of Biomedical Sciences, School of Public Health, University of Albany, Albany, NY, USA; d Department of Biosciences and Oral Diagnosis, Institute of Science and Technology, UNESP - Univ Estadual Paulista, Sao Jose dos Campos, Brazil; e Division of Infectious Diseases, Wadsworth Center, New York State Department of Health, Albany, NY, USA

**Keywords:** *Galleria mellonella*, infection model, invertebrate host, *Pseudogymnoascus destructans*, *Pseudogymnoascus pannorum*, survival curve, histopathology

## Abstract

Laboratory investigations of the pathogenesis of *Pseudogymnoascus destructans*, the fungal causal agent of bat White Nose Syndrome (WNS), presents unique challenges due to its growth requirements (4°-15°C) and a lack of infectivity in the current disease models. *Pseudogymnoascus pannorum* is the nearest fungal relative of *P. destructans* with wider psychrophilic – physiological growth range, and ability to cause rare skin infections in humans. Our broad objectives are to create the molecular toolkit for comparative study of *P. destructans* and *P. pannorum* pathogenesis. Towards these goals, we report the successful development of an invertebrate model in the greater wax moth *Galleria mellonella*. Both *P. destructans* and *P. pannorum* caused fatal disease in *G. mellonella* and elicited immune responses and histopathological changes consistent with the experimental disease.

## Introduction

White-nose syndrome (WNS) is an epizootic disease responsible for the deaths of more than 5 million bats in North America since its first discovery in 2006 [1,2]. The latest surveillance and modeling data suggest that WNS is likely to lead to the regional extinction of some bat species in the United States while the disease remains well-tolerated among the European bats [3,4]. *Pseudogymnoascus destructans* is the fungal causal agent of WNS [5]. *Pseudogymnoascus destructans* is a psychrophile, well-adapted to growing at 4°-15°C, and widely distributed in the caves and mines that serve as bat hibernacula [5–8]. Bats infected with *P. destructans* exhibit histopathological lesions on wings and other body parts, are frequently aroused from hibernation-induced torpor, and possibly succumb to WNS due to fat depletion and starvation [9–11]. Some North American bat species and bats elsewhere in the world do not suffer from WNS-associated fatality likely due to a protective immune response against *P. destructans* [4,12–14]

*P. destructans* is unique among known human and animal fungal pathogens for the manifestation of its virulence attributes at temperatures lower than the expected physiological range, which is a common trait of pathogenic fungi as detailed by Kohler et al. [15]. *P. pannorum* is the nearest fungal relative of *P. destructans*, which grows over psychrophilic – physiological temperatures, and causes rare skin infections in humans [5,16–18]. We have devoted efforts to create the molecular toolkit for the comparative studies of *P. destructans* and *P. pannorum* as there is no framework to investigate fungal pathogenesis at low temperatures. Towards this end, we earlier reported a comparison of the draft genomes of the two pathogens and created a transformation system for targeted gene disruption and live-cell imaging [19–21] . In the current report, an *in vivo* model is described using the wax moth *Galleria mellonella* to facilitate comparative pathogenesis studies. Both *P. destructans* and *P. pannorum* caused fatal disease in *G. mellonella* and elicited immune responses and histopathological changes that are the hallmark of experimental disease in this model system.

## Materials and methods

### Strains and media

*P. destructans* (M1379, PD251) and *P. pannorum* (M1372, PP1062) strains were routinely maintained on the yeast extract peptone dextrose (YPD) agar at 15°C and stored in 15% sterile glycerol at −70°C. Potato dextrose agar (PDA; Difco) and Sabouraud dextrose agar were used for growth at 5°C or 15°C as described previously [20].

### G. mellonella *survival studies*

Inocula were prepared by collecting *P. destructans* and *P. pannorum* conidia from cultures grown in PDA agar at 15°C for one-week, harvested by gently scraping the fungal growth, suspending in phosphate buffered saline (PBS) and passing the suspension through a 27G needle to separate conidia. Conidia were washed twice with PBS by centrifugation and counted with a hemocytometer to determine the fungal cell concentration, and plated on culture plates to determine colony forming units (CFU). *G. mellonella* larvae (Vanderhorst Wholesale, St. Marys, OH) at their final instar stage were inoculated with 5 × 10^5^ or 1 × 10^6^ CFU of *P. destructans* or *P. pannorum* suspended in PBS. Each infection group contained 16 randomly chosen larvae of the appropriate weight (330 ± 25 mg). The inoculum was injected in a 10 μl volume directly to the last left pro-leg using a Hamilton syringe [22]. Larvae were incubated at 5°C and 15°C, and the number of dead larvae was scored daily. Killing curves were plotted, and statistical analysis was performed by the Kaplan-Meier method using STATA 6 statistical software (Stata). Killing curves were performed in duplicate, and representative graphs were reported.

### G. mellonella *hemocyte density*

In an experiment subsequent to survival studies, the larvae were injected with the fungi at concentrations of 5 × 10^5^ CFU/larvae or 1 × 10^6^ CFU/larvae at the last left pro-leg then incubated at 15°C. Hemocytes were collected from the hemocoel at 24 h post-injection. Larvae were punctured with a number 11 blade scalpel and bled into tubes containing cold, sterile insect physiologic saline (IPS) (150 mM sodium chloride; 5 mM potassium chloride; 100 mM Tris–hydrochloride, pH 6.9 with 10 mM EDTA, and 30 mM sodium citrate), pooling four larvae for each group. The hemocytes were enumerated with the aid of a hemocytometer. However, we did not differentiate between the six types of hemocytes (prohemocytes, coagulocytes, spherulocytes, oenocytoids, plasmatocytes, and granulocytes). Results were averaged from four replicates, and a Student’s t-test was used to compare groups, *P *< 0.05 was considered significant.

### Histopathology

Infected larvae were fixed in buffered formalin at 4°C for 48-hours, and embedded in paraffin. Paraffin blocks were sectioned and stained with H&E and Periodic acid-Schiff (PAS). Blind histopathological evaluation was performed on 8–9 full-length sagittal sections of larvae in each group.

## Results and discussion

**Figure 1.**
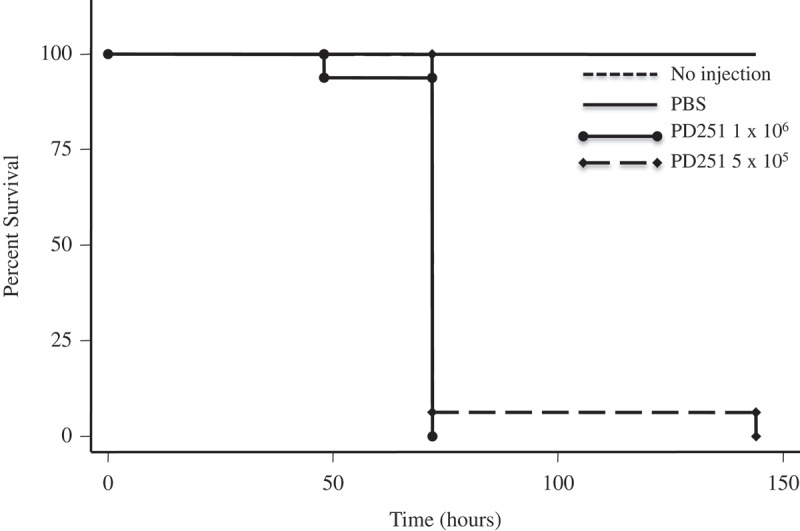
*P. destructans* reduced survival of the *G. mellonella* infection host. Larvae infected and incubated at 15°C experienced decreased survival compared to larvae injected with PBS per dosage indicated.

**Figure 2.**
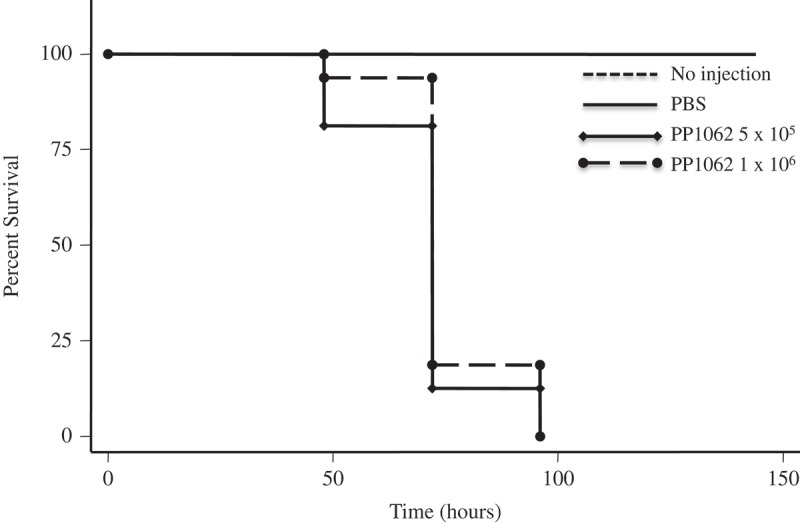
*P. pannorum* reduced survival of the *G. mellonella* infection host. Larvae infected and incubated at 15°C experienced decreased survival compared to larvae injected with PBS per dosage indicated.

*Pseudogymnoascus destructans* and *P. pannorum* caused temperature and dose-dependent mortality in *G. mellonella. P. destructans*- and *P. pannorum*-injected *G. mellonella* suffered significant mortality at 15°C compared to larvae injected with PBS, based on assessment with a Kaplan-Meier survival plot (*P *< 0.001). This mortality pattern was consistent with 5 × 10^5^ CFU and 1 × 10^6^ CFU within the 144-hour evaluation period (Figures 1–2). Mortality of 50% was reached as early as 75-hours post-inoculation for the infected groups. At 5°C, there was no impact on the larvae survival by different dosage of inoculum of both pathogens (data not shown).

**Figure 3.**
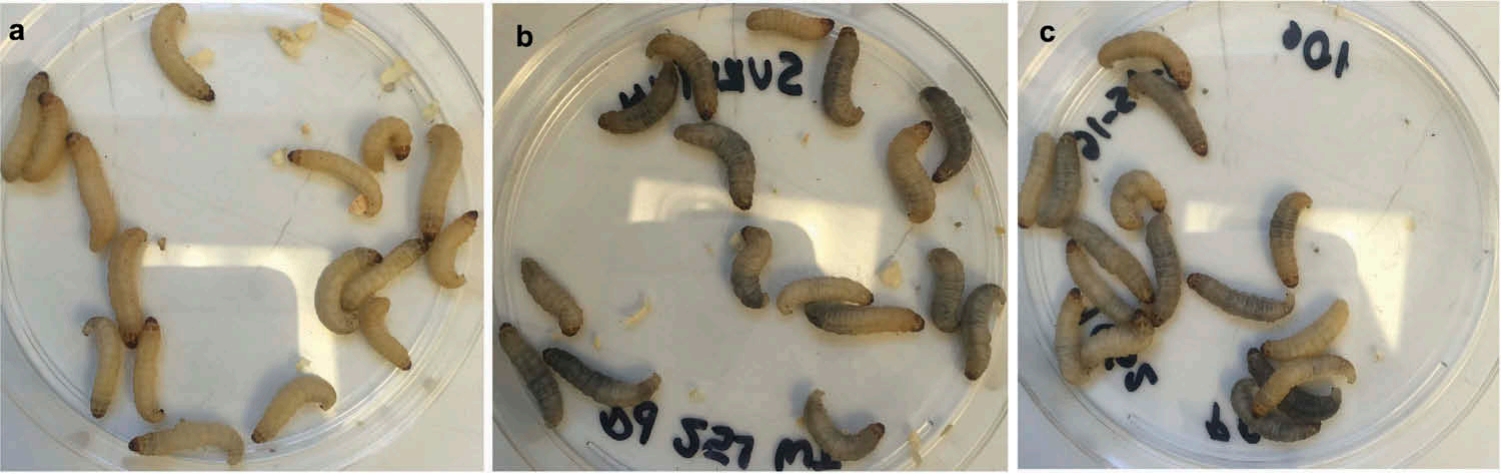
*P. destructans* and *P. pannorum* infection caused melanization in *G. mellonella*. a) PBS-injected control larvae, b) Larvae injected with 1 × 10^6^ *P. destructans*, c) larvae injected with 1 × 10^6^ *P. pannorum*. All photographs were taken approximately 60-min after injections.

**Figure 4.**
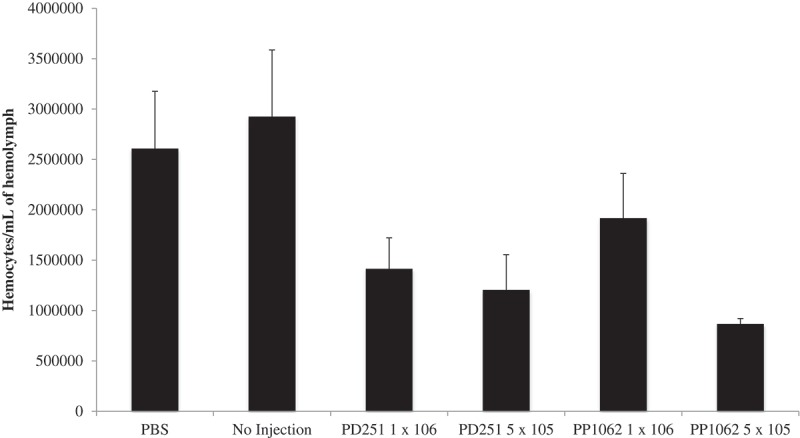
Larvae infected with both *Pseudogymnaoscus* species exhibited a decrease in the hemocyte density. The hemocyte density within the hemolymph was reduced significantly compared to larvae injected with PBS (**P < 0.01). The legends include *P. destructans* (PD251-M1379) and *P. pannorum* (PD1062-M1372).

Upon visual inspection of larvae, we observed melanization induced within larvae infected with either *Pseudogymnoascus* species (Figure 3), suggesting recognition of the pathogen by the host immune system. The larvae exhibited melanization at the injection site; the pigmentation spread throughout the body, and it was retained during the infection until death. Infection with *P. destructans* and *P. pannorum* resulted in reduced hemocyte density within the hemolymph compared to larvae injected with PBS (Figure 4) (*P *< 0.01). The reduction in hemocyte density was significant with both 5 × 10^5^ CFU (*P *< 0.05) and 1 × 10^6^ CFU (*P *< 0.05) of *P. destructans*. A significant reduction in hemocyte density was also observed with 5 × 10^5^ CFU (*P *< 0.05) of *P. pannorum* while injection of higher dosage (1x10^6^ CFU) did not result in significant reduction (*P *> 0.05).

**Figure 5.**
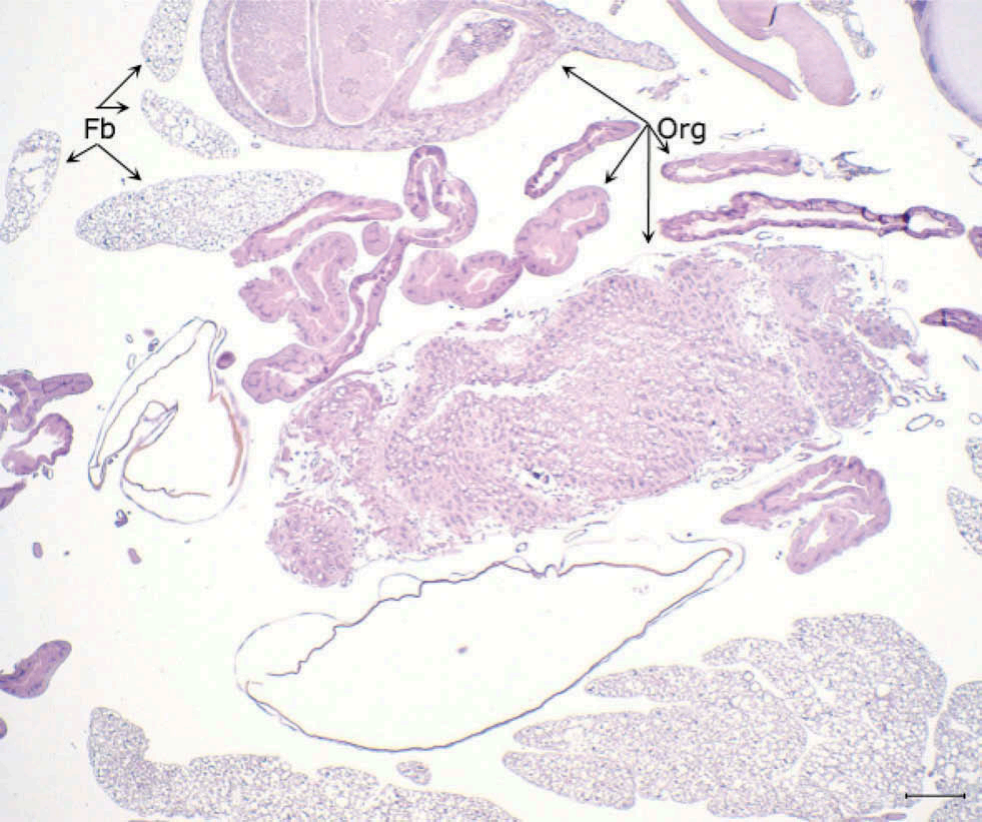
Histopathology of *G. mellonella inoculated* with PBS (PAS stain). Note cross-sections of organelles (Org), fat bodies (Fb), and lack of melanization in the hemocoel cavity (5x; scale 100 µm).

**Figure 6.**
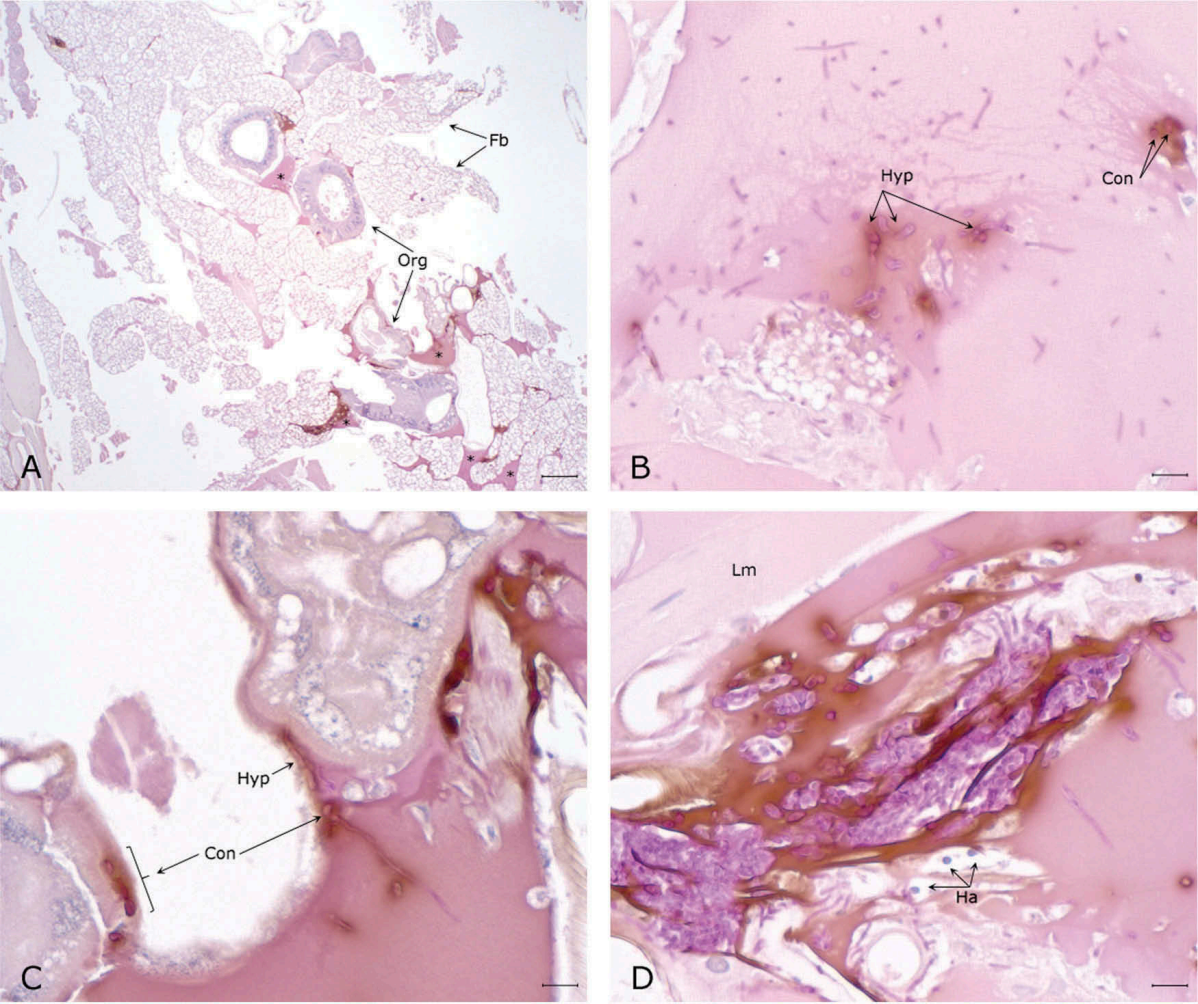
Histopathology of *G. mellonella* infected with *P. destructans* and kept at 5°C. A. Throughout the hemocoel there are numerous lakes of dense eosinophilic material with melanin deposition (*) around organelles (Org) and expanding in the fat body (Fb) (5x; scale 100 µm). B. Higher magnification of hemolymph lake with numerous PAS-positive hyphae (Hyp) and conidia (Con) with concomitant mild melanization. C. Hyphae and conidia on the surface of organelles eliciting moderate melanization. D. Densely packed PAS-positive hyphae and conidia expanding between longitudinal muscle fibers (Lm) eliciting marked melanization and the infiltration of hemocytes (Ha). (50x; scale 10 µm).

**Figure 7.**
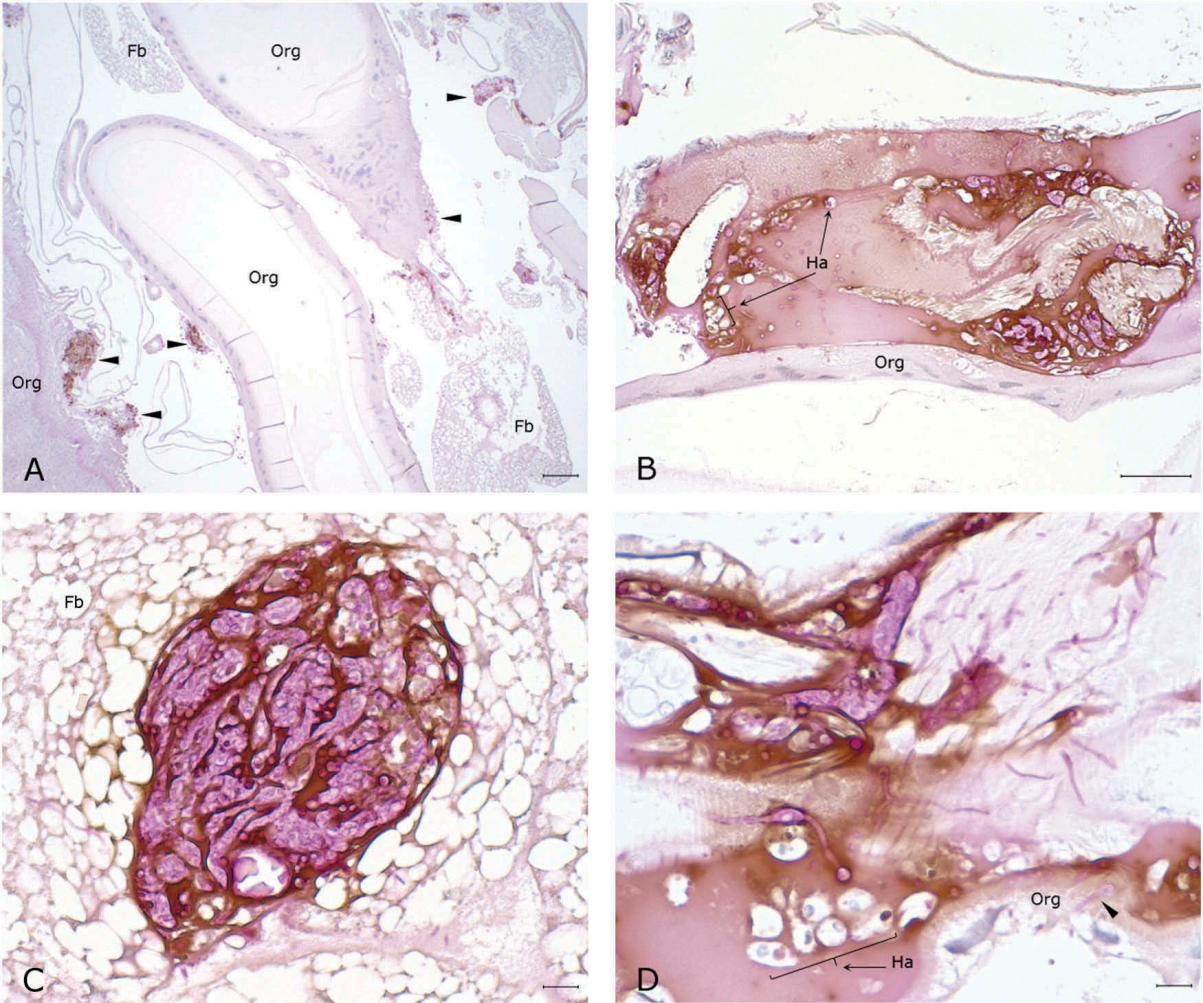
Histopathology *G. mellonella* infected with *P. pannorum* and kept at 5°C. A. Numerous nodules and aggregates of melanin deposition (arrow heads) on the wall of organelles (Org) and fat body (Fb) (5x; scale 100 µm). B-D. Note PAS-positive fungal elements and infiltration of hemocytes (Ha) in melanin nodules. D. Hyphae invading organelle wall (arrow head). (50x; scale 10 µm).

**Figure 8.**
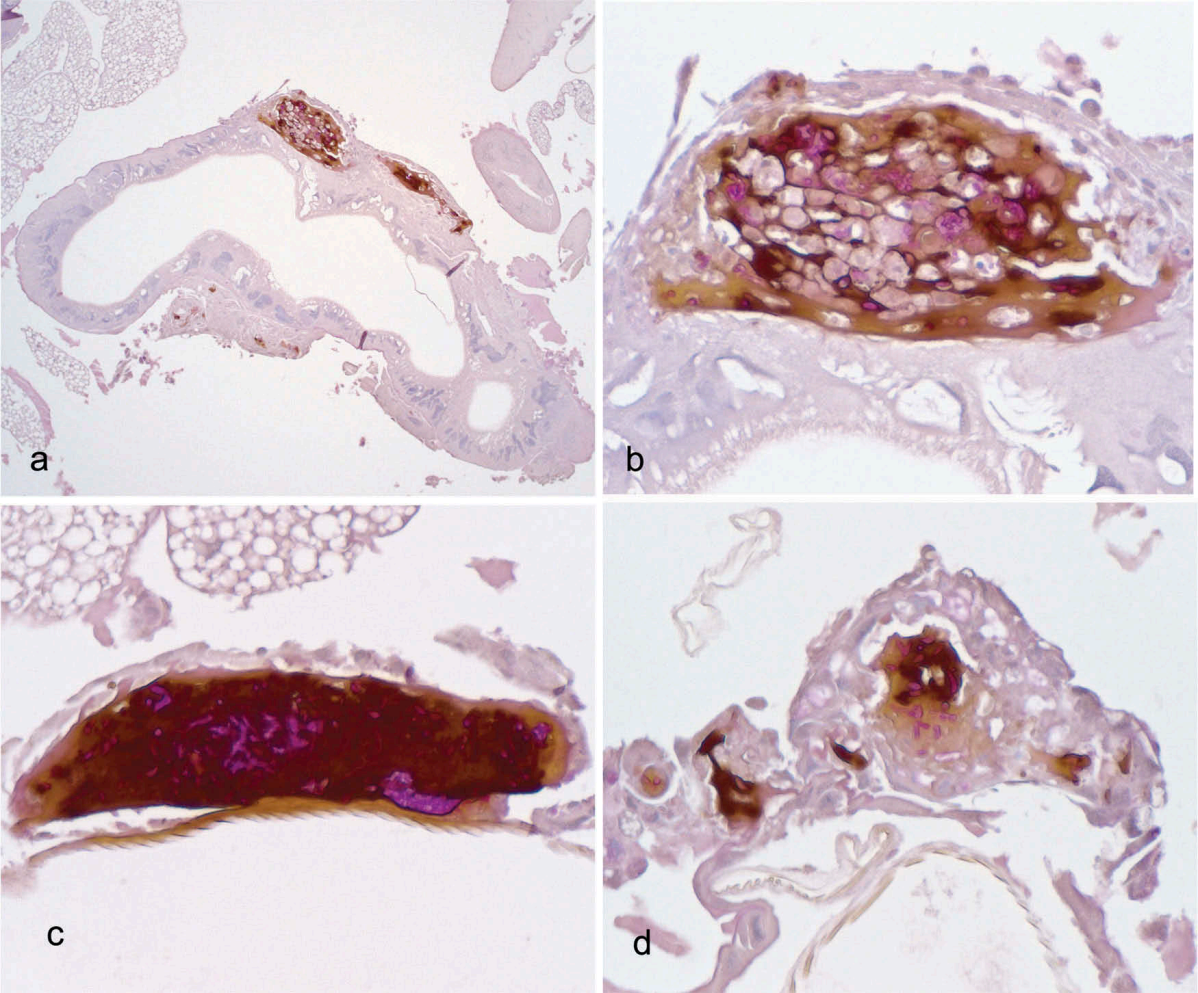
Histopathology of *G. mellonella* infected with *P. destructans* and kept at 15°C. A. Granuloma-like hemocyte nodules (arrow heads) with prominent melanin deposition expanding in the wall of organelle (Org) (10x; scale 100 µm). B. Higher magnification of melanized granuloma-like nodule, note numerous PAS-positive fungal elements admixed with hemocytes (*). C-D. Granuloma-like nodule with prominent PAS-positive fungal elements and marked melanin deposition on the wall of organelles (arrow heads). (50x; scale 10 µm).

**Figure 9.**
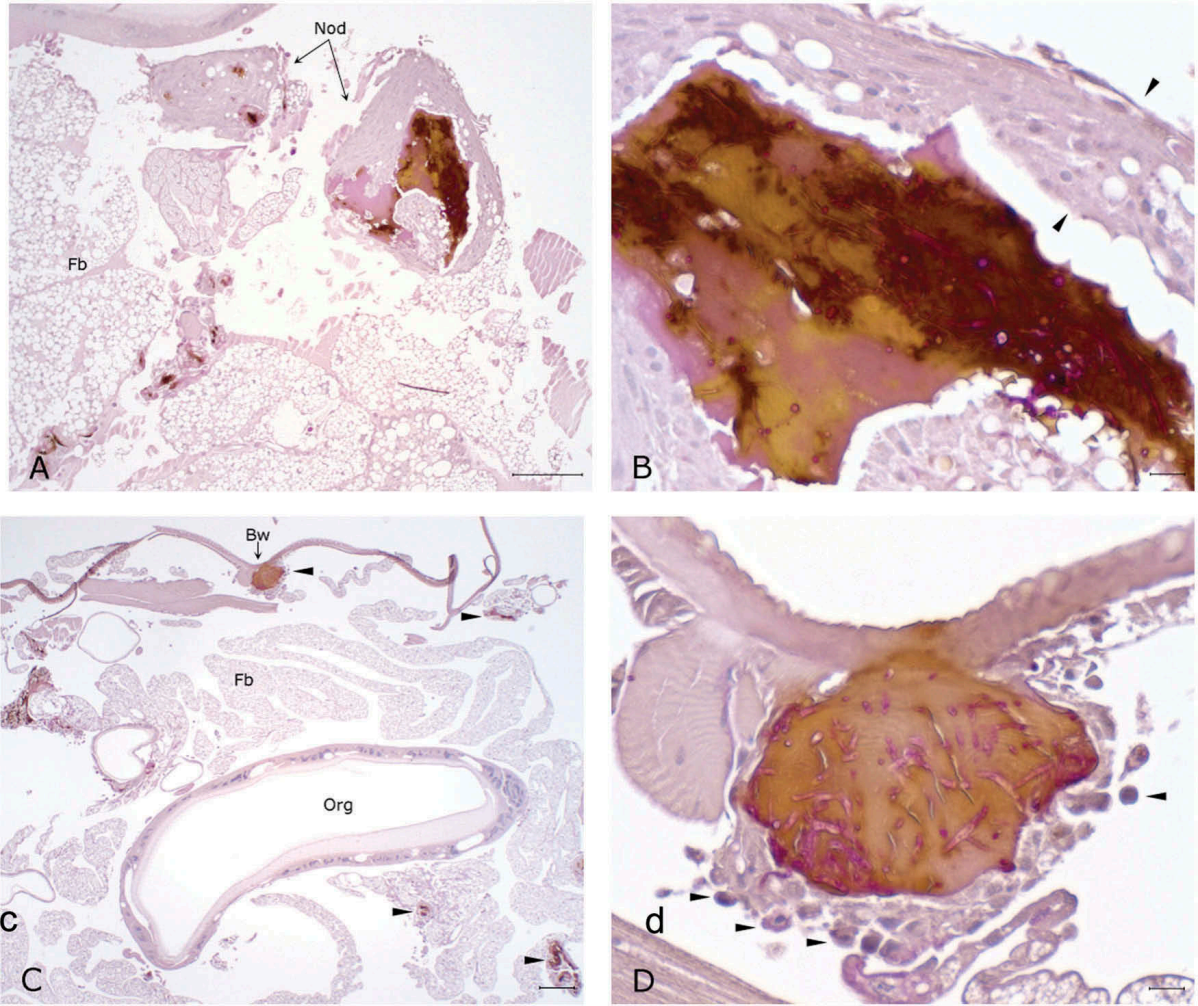
Histopathology of *G. mellonella* infected with *P. pannorum* and kept at 15°C. A. Large granuloma-like hemocyte nodules (Nod) with prominent melanin deposition in the hemocoel cavity (10x; scale 100 µm). B. Higher magnification of granuloma-like nodule. Note thick capsule of round-to-spindle cells (arrow heads) surrounding PAS-positive fungal elements and prominent melanin deposits (50x; scale 10 µm). C. Overview of hemocoel cavity depicting numerous melanized granuloma-like nodules (arrow heads), including in the body wall (Bw) (5x; scale 100 µm). D. Higher magnification of granuloma-like nodule expanding in the body wall. Note prominent PAS-positive hyphae and conidia eliciting melanization and infiltration of hemocytes (arrow heads) (50x; scale 10 µm).

The histopathological examination of the hemocoel of uninfected sham inoculated (PBS) larvae showed normal organelles and fat bodies, and lack of melanization in the hemocoel cavity (Figure 5). All larvae inoculated with *P. detructans* or *P. pannorum* showed evidence of fungal dissemination throughout the hemocoel. Variable numbers and sizes of melanized spots were observed, which represented immune-competent hemocytes surrounding entrapped fungal spores or hyphae. The larvae inoculated with either fungus and housed at 5°C, had numerous clusters of hemocytes admixed with hemolymph, melanin, conidia, and hyphae throughout the hemocoel (Figures 6 – 7). These clusters were less organized in the larva inoculated with *P. destructans* and mostly expanding in the fat body and coelomic space (Figure 6). In the *P. pannorum* inoculated larvae, there were numerous loosely and well-defined hemocyte nodules throughout the hemocoel (Figure 7). In both groups, the hemocyte nodules consisted of conidia and hyphae surrounded by prominent melanin deposition and scattered hemocytes (Figures 6–7). The histopathological changes were more severe in the larvae inoculated with either *P. destructans* or *P. pannorum* and housed at 15°C (Figures 8 – 9). Additionally, there were subtle differences in the severity of the immune response and dissemination. The *P. destructans* inoculated larvae had numerous granuloma-like hemocyte nodules throughout the hemocoel, fat body, and expanding within the wall of organelles containing round-to-spindloid hemocytes encapsulating fungal elements and abundant melanin (Figure 8). Similar immune response and distribution were observed in the larva inoculated with *P. pannorum* with wider dissemination of the fungal elements into the body wall and cuticle (Figure 9).

Our novel findings establish a tractable experimental system to study host-pathogen interactions of psychrophilic fungi *P. destructans* and *P. pannorum*. Many earlier publications have described the wax moth model as a preferred *in vivo* system for the study of human, insect, and fish pathogenic fungi [23–27]. Our findings are novel as we took advantage of the *G. mellonella* ability to survive in a wide range of temperature to conduct the experiments at low temperature. In contrast, other studies maintained the infected *G. mellonella* larvae at temperatures ranging from 25° to 37°C. The present study provided proof that the *G. mellonella* experimental system is amenable to an extended temperature range from 5°C to 37°C. It is well-known that temperature affects *G. mellonella* immune response to pathogens; however, the relevant previous studies focused on the effects of pre-incubation periods as they related to the immune responses [28,29]. Importantly, the current study differs from earlier publication in that the larvae were maintained at room temperature until infection and were not placed at 5°C and 15°C until after infection. It is likely that in this case, the lower temperature was conducive both for the growth of the fungal pathogens and *G. mellonella*. The fact that robust immune response is invoked in *G. mellonella* kept at 4°C might have contributed to the lack of *P. destructans*- and *P. pannorum*-induced mortality in this study [27,29]

The decrease in hemocyte density after *P. destructans* and *P. pannorum* infections also held true at low temperatures as reported for the higher temperatures in other studies. The hemocyte decline fulfilled an additional correlation of experimental disease as postulated by Bergin and colleagues [30]. The decline in hemocyte count was not dose-dependent likely because the experiments were performed with different batches of larvae. The observed melanin in the infected larvae suggested that the insect host mounted a robust albeit ineffective immune response in the form of phenoloxidase activation and resulting melanin production [24].

*Galleria mellonella* models have become invaluable to study cellular and humoral immune responses against pathogens [31–33], epigenetic influences on the evolution of virulence [32,34,35], and for development and testing antimicrobials [36–39]. It is relevant to add that the complete genomes of *P. destructans* and *G. mellonella* have become available recently [40,41]. Thus, *G. mellonella* – *Pseudogymnoascus* pathosystem is now ready as a tractable model for the fungal gene–phenotype studies as well as for the high-throughput screens in search for effective antifungal agents.

## Abbreviations

colony forming unit: CFU;
insect physiologic saline: IPS;
phosphate buffered saline: PBS
